# Insights about exosomal circular RNAs as novel biomarkers and therapeutic targets for hepatocellular carcinoma

**DOI:** 10.3389/fphar.2024.1466424

**Published:** 2024-10-09

**Authors:** Haiyan Zhang, Shanshan Pei, Jiaxuan Li, Jiajie Zhu, Hongyu Li, Guangshang Wu, Ruiqi Weng, Ruyi Chen, Zhongbiao Fang, Jingbo Sun, Keda Chen

**Affiliations:** ^1^ Key Laboratory of Artificial Organs and Computational Medicine of Zhejiang Province, Shulan International Medical College, Zhejiang Shuren University, Hangzhou, China; ^2^ Zhejiang Chinese Medical University, Shuren College, Hangzhou, China; ^3^ School of Pharmacy, Beihua University, Jilin, China

**Keywords:** hepatocellular carcinoma, exosomes, circRNA, biomarker, cancer therapy

## Abstract

One of the most prevalent pathological types of Primary Liver Cancer (PLC) is the Hepatocellular Carcinoma (HCC) poses a global health issue. The high recurrence and metastasis rate of HCC, coupled with a low 5-year survival rate, result in a bleak prognosis. Exosomes, small extracellular vesicles released by various cells, contain diverse non-coding RNA molecules, including circular RNAs (circRNAs), which play a significant role in intercellular communication and can impact HCC progression. Studies have revealed the potential clinical applications of exosomal circRNAs as biomarkers and therapeutic targets for HCC. These circRNAs can be transferred via exosomes to nearby non-cancerous cells, thereby regulating HCC progression and influencing malignant phenotypes, such as cell proliferation, invasion, metastasis, and drug resistance. This review provides a comprehensive overview of the identified exosomal circRNAs, highlighting their potential as non-invasive biomarkers for HCC, and suggesting new perspectives for HCC diagnosis and treatment. The circRNA from exosomal organelles promotes metastasis and immune scape because of their unique chirality which is different from the Biomolecular Homochirality.

## 1 Introduction

Primary liver cancer (PLC), which is one of the most prevalent gastrointestinal tumors globally, ranks fifth in terms of incidence worldwide and is among the top three causes of cancer-related deaths (Sung et al., 2021). In 2020, There were over 900,000 new cases and about 830,000 deaths globally (Sung et al., 2021), with hepatocellular carcinoma (HCC) accounting for approximately 75%–90% of PLC cases (Center and Jemal, 2011). The major risk factors for HCC include chronic infection with hepatitis viruses, such as hepatitis B virus (HBV) or hepatitis C virus (HCV), obesity, aflatoxin exposure, and excessive alcohol consumption (EATSLEORTC, 2004; Heilig et al., 2004; Marcon et al., 2018). Alpha-fetoprotein (AFP) is the most commonly used serum biomarker for HCC diagnosis in clinical practice; however, it has poor sensitivity and specificity for early diagnosis (Omar et al., 2023). Clinical studies have found that approximately 40% of patients with HCC are negative for AFP (Daniele et al., 2004), and even lower positive rates are observed in postoperative patients with metastatic HCC (Huang et al., 2020). AFP combined with ultrasound can be used for early HCC screening; however, ultrasound diagnosis relies heavily on the operator’s subjective judgment and might result in a low detection rate for early HCC (Hartke et al., 2017). In addition, des-gamma-carboxy prothrombin (DCP) and Lens culinaris agglutinin-reactive fraction of alpha-fetoprotein (AFP-L3) are also used to diagnose early HCC, especially in patients with negative imaging results; their sensitivity is low (Sterling et al., 2009). For the early HCC, the means of diagnosis recommended by all clinical guidelines remains unsatisfactory (Wang and Wei, 2020).

The progression of HCC correlates negatively with prognosis. Radical treatment options, including surgical resection, radiofrequency ablation (RFA), and liver transplantation, are suitable for only approximately one-third of early-stage patients, with a 5-year survival rate exceeding 50% (2012) The majority of advanced-stage patients undergo non-curative treatments, such as transarterial chemoembolization (TACE), chemotherapy, immunotherapy, and targeted therapy (Anwanwan et al., 2020). However, these treatments are associated with high rates of recurrence and metastasis (Allaire et al., 2020), leading to a 5-year survival rate of only about 18% (Jiang et al., 2019). Moreover, increased resistance to therapeutic drugs, such as immune checkpoint inhibitors, sorafenib, and levatinib, further decreases the efficacy of treatment for patients with advanced-stage HCC (Anwanwan et al., 2020). Multi-drug combination therapy and special drug delivery technology, such as nanotechnology, aim to reduce drug resistance and improve curative effects (Xia et al., 2017); however, the incidence of HCC is still increasing (Sung et al., 2021). Further research is needed to find better ways to diagnose and treat HCC.

With the advances in high-throughput chips and second-generation sequencing technologies, an increasing number of non-coding RNAs have been discovered. Circular RNAs (circRNAs) are non-coding RNA molecules that form a closed loop via covalent binding, which can regulate gene expression at the transcriptional and post-transcriptional levels, acting as miRNA sponges, encoding peptides or proteins, and forming stable RNA-protein complexes to regulate downstream biological processes (Meng et al., 2017; Kos et al., 1986). CircRNAs are stable, conserved, abundant, and tissue or stage-specific, allowing them to play a crucial role in various diseases, especially in tumors (Memczak et al., 2013; Meng et al., 2017; Chen and Shan, 2021). Studies found that circRNAs loaded in exosomes can promote metastasis between tumor cells and non-tumor cells, enhance or inhibit tumor progression, and can be detected using liquid biopsies (Pang et al., 2020). Exosomal *circSHKBP1* promotes gastric cancer progression by regulating the miR-582-3p/Hu antigen R (HUR)/vascular endothelial growth factor (VEGF) pathway) (Xie et al., 2020). Exosomal *ciRS-122* induced an increase of chemotherapy resistance in colorectal cancer, and exosomal has-circ-0001380 affects the occurrence and development of HCC (Wu et al., 2022). In recent years, research around exosomal circRNAs has gradually increased, and these molecules have emerged as attractive candidates for the early diagnosis and treatment of HCC. In this review, we summarize the exosomal circRNAs that might be developed to improve the prognosis of HCC.

## 2 The characteristics of exosomes

**FIGURE 1:**
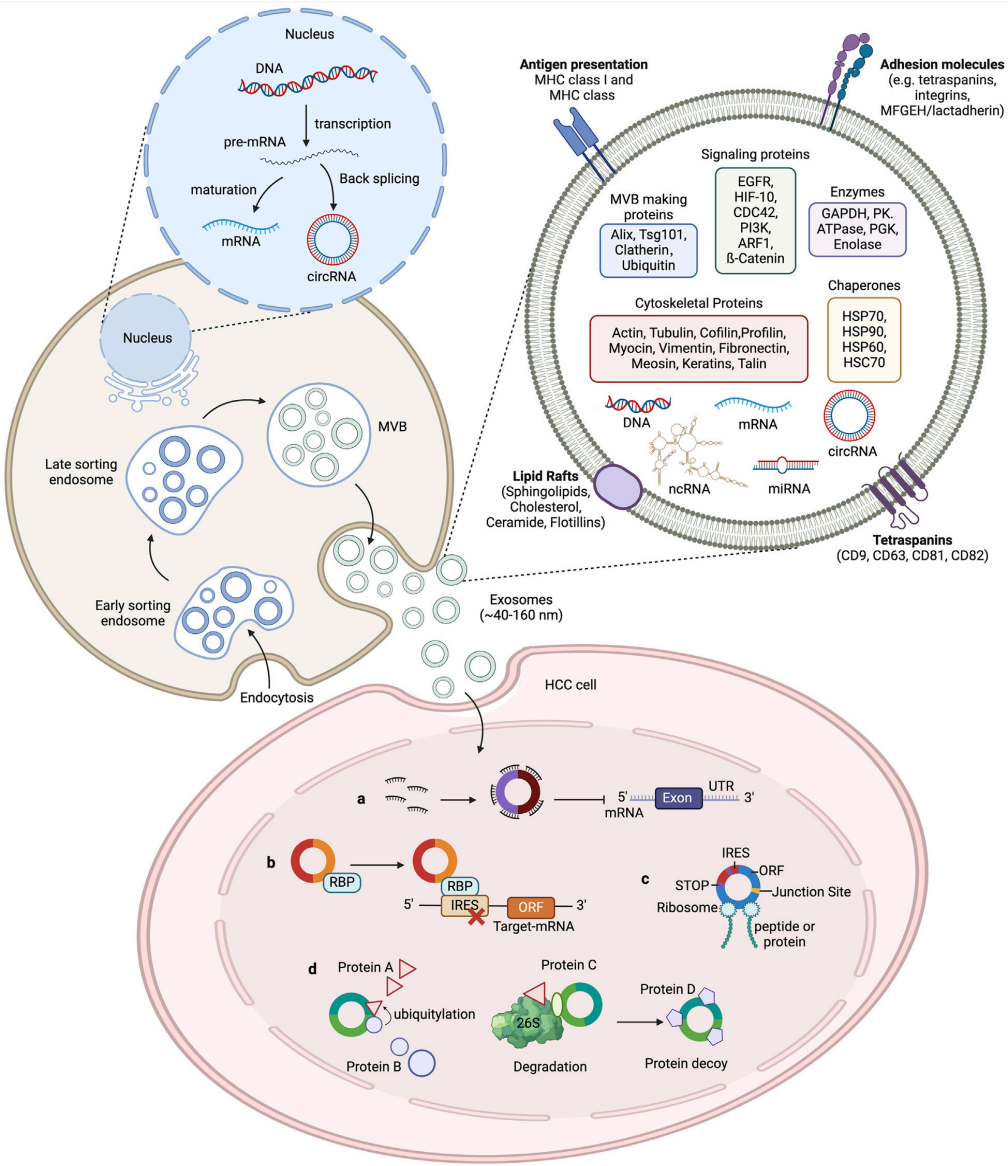
The biogenesis of exosomes and the function of circRNAs in HCC. The cytoplasmic membrane internalizes extracellular components through endocytosis, forming early endosomes. During endosome maturation, they are classified as intraluminal vesicles (ILV) within the endosome. The endosomal membrane further folds to form multivesicular bodies (MVB) that contain ILVs. Selectively or passively, various cellular contents such as RNA (including mRNA, circRNA, and other ncRNAs), DNA, and lipids are integrated into the vesicles. Subsequently, MVBs can either fuse with lysosomes for degradation or merge with the plasma membrane to release the ILVs, now called exosomes, into the extracellular fluid, where they perform their physiological functions. Exosomal circRNAs play an important role in tumour development. CircRNAs regulate the transcription of HCC target genes by acting as a microRNA sponges to impair microRNA function and protect target mRNAs from degradation. CircRNAs also bind to RNA-binding proteins (RBPs) to regulate the expression of relevant genes. CircRNAs interact with proteins to affect their structure and activity. Some circRNAs contain an internal ribosome entry site (IRES) element, in which the AUG site serves as a template for coding peptides or proteins.

Exosomes are small extracellular vesicles (EVs) enclosed by a lipid bilayer and measuring between 40 and 120 nm in diameter (S et al., 2013). They were initially discovered in sheep reticulocytes in 1983 and can be released by various cells under both normal and pathological conditions (Harding et al., 1983).^[1]^ Exosomes contain DNA, proteins, lipids, circRNAs, and other functional molecules, and can remain stable in bodily fluids such as blood, urine, cerebrospinal fluid, amniotic fluid, and breast milk (Xu et al., 2016). The process of exosome formation begins with the formation of vesicles via endocytosis from the cell membrane, followed by the inward budding of vesicles to create multivesicular bodies (MVBs). Eventually, the MVBs fuse with the cytoplasmic membrane, thereby releasing exosomes into the extracellular fluid (Kowal et al., 2014). Exosomes can transfer certain functional molecules, such as proteins and circRNAs, between different cells to mediate cell-to-cell communication, which regulates protein synthesis, cell growth and differentiation, antiviral activity, and numerous other physiological and pathological activities (Wang et al., 2019b). Exosomes mediate cell communication through four main mechanisms: (1) The binding of exosomal membrane proteins to target cells, which activates intracellular signaling pathways; (2) the delivery of functional proteins or infectious particles to recipient cells; (3) the transfer of receptors between cells; and (4) the transfer of genetic information through mRNA, miRNA, circRNA, or transcription factors between cells (as shown in Figure 1). (Masyuk et al., 2013).

Exosomes are involved in tumor initiation, immune escape, drug resistance, and drug delivery, making them highly valuable in the diagnosis, prognosis, and treatment of tumors (Wu et al., 2022; Kalluri and LeBleu, 2020). In addition, because of their small size and ability to evade elimination by other cells, exosomes can serve as carriers for drugs and functional RNAs (2004) Nanotechnology-based delivery of exosomes as carriers for drugs and functional RNAs to tumor cells has shown great potential for precise tumor treatment without causing adverse reactions, thereby offering promising clinical applications (Ha et al., 2016).

## 3 The function of exosomal circRNAs

In the human genome sequence, approximately 98% of genes do not encode proteins (2004) Non-coding RNAs can be classified into linear and circular forms based on their structural characteristics (Yu and Shan, 2016). Linear non-coding RNAs can be further categorized into small non-coding RNAs and long non-coding RNAs (lncRNAs). MicroRNAs, siRNAs, and small nucleolar RNAs (snoRNAs) are examples of small non-coding RNAs, with microRNAs (miRNAs) being the most extensively studied (Hombach and Kretz, 2016). The presence of circular RNAs (circRNAs) was initially discovered in RNA viruses, such as retroviruses and hepatitis viruses, in 1976 (Sanger et al., 1976). It was later observed that circRNAs are also produced in eukaryotes during transcription. Initially regarded as “byproducts” of transcription because of their lack of protein-coding ability, circRNAs were not extensively investigated by researchers (Cocquerelle et al., 1993). CircRNAs are typically formed through the reverse splicing of precursor mRNAs, resulting in a single-stranded closed-loop structure held together by covalent bonds (Chen and Yang, 2015). Based on their origin, circRNAs are classified into four categories: exonic circRNAs, intronic circRNAs, circRNAs formed by introns and exons, and intergenic circRNAs (Wang et al., 2016). Compared with linear RNAs, circular RNAs possess greater resistance to degradation by nucleases and RNA enzymes because of the absence of free 5′ cap structures and 3′ poly(A) tails (Yu and Shan, 2016). CircRNAs containing miRNA binding sites (MREs) can directly regulate gene expression by binding to miRNAs and indirectly influence the expression levels of downstream target genes by interacting with RNA-binding proteins and affecting mRNA stability (Feng et al., 2019). Moreover, CircRNAs with RNA protein binding sites can interact with proteins, and serve as protein decoys, scaffolds and recruiters to interact with one or more proteins, protein conformations are influenced to alter protein-protein interactions, but the mechanisms need to be further studied (Zhou et al., 2020). Differential expression of circRNAs has been observed in various malignant tumor cells and tissues and has shown significant correlations with tumor staging and prognosis, implying their potential involvement in tumor initiation and progression (Wang et al., 2018).

The mechanisms of circRNA action vary depending on their subcellular location. CircRNAs derived from exonic regions are predominantly found in the cytoplasm and primarily function as miRNA sponges, thereby modulating the expression of downstream target genes. An example is *has-circ-0000519*, which is enriched in the cytoplasm and promotes HCC angiogenesis by influencing the miR-1296/E2F transcription factor 7 (E2F7) axis (Liu et al., 2023b). Conversely, circRNAs localized in the nucleus typically originate from introns or consist of both exonic and intronic sequences. They regulate gene expression at the transcriptional or post-transcriptional level (Memczak et al., 2013). Exosomal *circ-ZNF652* is upregulated in the serum and cells of patients with HCC, promoting HCC cell proliferation, invasion, and metastasis through targeting the miR-29a-3p/guanylyl cyclase domain containing 1 (GUCD1) axis (Li et al., 2020). Numerous studies have demonstrated the oncogenic or tumor suppressive roles of circRNAs in HCC development, with some circRNAs being closely associated with HCC prognosis. In malignant tumors like HCC, circRNAs often act as competitive endogenous RNAs (ceRNAs) by interacting with miRNAs in a circRNA/miRNA/mRNA axis to regulate downstream gene expression (Ng et al., 2018). Additionally, circRNAs can interact with transcription factors, mediating their role in epithelial-mesenchymal transition (EMT) and promoting HCC progression (Xu et al., 2020b).

In 2015, circRNAs were first discovered in exosomes, and subsequent research revealed that circulating exogenous circRNAs could differentiate non-patients from patients based on their expression levels (Li et al., 2015). Further investigations confirmed the stable existence and wide enrichment of circRNAs in exosomes, particularly those derived from tumor cells, thus exosomal circRNAs have been identified as potential biomarkers for tumor diagnosis and prognosis (Li et al., 2015). In malignant tumors, most exosomal circRNAs function as miRNA sponges, exerting oncogenic or tumor suppressive effects. For instance, exosomal *circ-PDE8A*, derived from pancreatic ductal adenocarcinoma, regulates metastasis-associated in colon cancer 1 (MACC1) expression by binding to miR-338, stimulating cancer cell invasive growth via the MACC/MET/extracellular regulated kinase (ERK) or protein kinase B (AKT) pathway, and the expression levels of exosomal circRNAs closely correlate with patient prognosis (Li et al., 2018). Exosomal *circLPAR1* inhibits bromodomain containing 4 (BRD4) expression through its interaction with Methyltransferase 3, N6adenosine-methyltransferase complex catalytic subunit (METTL3)-eukaryotic translation initiation factor 3 subunit H (eIF3h), playing a crucial role in the diagnosis and treatment of colorectal cancer (Zheng et al., 2022).

## 4 The mechanisms of exosomal circRNAs in HCC

The tumor microenvironment (TME) is formed through the interaction of various cells, including both tumor and non-tumor cells (Hanahan and Coussens, 2012). It consists of extracellular matrix components (ECM), stromal cells (such as cancer-associated fibroblasts, mesenchymal stem cells, pericytes, and endothelial cells), different immune cells (including T and B lymphocytes, natural killer (NK) cells, regulatory T cells, and tumor-associated macrophages) (Xiao and Yu, 2021). The TME plays a crucial role in tumor progression, and understanding the TME and its intricate interactions opens up new possibilities for anti-cancer treatments, making significant contributions to improving treatment efficacy and reducing mortality rates (Xiao and Yu, 2021). Recent studies have highlighted the role of circRNAs in regulating the TME (Zhang et al., 2020b). CircRNAs are abundant and stable in exosomes derived from HCC cells, which can be secreted into the TME and delivered to different target cells through systemic circulation, affecting malignant characteristics such as tumor proliferation, metastasis, invasion, angiogenesis, immune response, and drug resistance (Li et al., 2015). Accumulating studies have shown that exosomes can remodel the TME to induce an inhibitory immune microenvironment to promote the progression of HCC (Li et al., 2023). Thus, exosomal circRNAs play an essential role in regulating communication between cancer cells and their surrounding cells in the TME, either promoting or inhibiting the invasion and metastasis of HCC. In addition, exosomal circRNAs have emerged as a promising therapeutic strategy for malignant tumors, including HCC, utilizing the exosomal RNA delivery system; the use of RNA-based targeted therapy has shown potential as a novel treatment modality for cancer (Zhang et al., 2016). Thus, circRNAs are expected to serve as effective biomarkers for the diagnosis, treatment, and prognosis of HCC, particularly those found in exosomes.

The study of exosomal circRNAs can help predict and assess the early progression of HCC without the need for invasive clinical procedures, which is a significant advantage for clinical application. Recent research has shown that exosomal *hsa-circ-0028861* and *hsa-circ-0070396* have excellent diagnostic value in HBV-related HCC. They can effectively differentiate patients with HCC from those with chronic hepatitis B and cirrhosis, which is of great significance in improving the diagnosis rate and prognosis of early-stage HCC (Wang et al., 2021b; Lyu et al., 2021). The combination of serum AFP and exosomal circRNA might further improve the early diagnosis rate of HCC and have critical clinical significance in the diagnosis of HBV-derived HCC.

### 4.1 The role of exosomal circRNAs from other cells in HCC

The exosomal circRNAs derived from HCC cells and adjacent non-cancerous cells might have different mechanisms of action (Shi et al., 2020). *Hsa-circ-0051443*, which is highly expressed in normal cells, can be transferred to HCC cells via exosomes. It can competitively bind to miR-331-3p leading to decreased expression of BCL2 antagonist/killer 1(BAK1), thus inhibiting HCC cell growth and promoting apoptosis. Moreover, the level of *hsa-circ-0051443* could differentiate between patients with HCC and non-HCC individuals, with an area under the curve (AUC) of 0.8089 (Chen et al., 2020). However, further clinical validation is required for accurate diagnosis.

Macrophages play a well-established role in HCC, and understanding their mechanism is essential for HCC treatment (Tian et al., 2019). Recent studies have demonstrated that exosomes derived from macrophages with high expression of recombination signal-binding protein Jk (RBPJ) can effectively inhibit the progression of HCC, and further investigation has revealed that exosomes carry *hsa-circ-0004658*, which is abundantly expressed in macrophages overexpressing RBPJ. This circRNA sponges miR-499b-5p, leading to an upregulation of junctional adhesion molecule 3 (JAM3). As a result, HCC cell proliferation is inhibited, and apoptosis is promoted (Zhang et al., 2022a). However, the results still need to be validated in a clinical cohort. In addition, it should be noted that not all exosomal circRNAs derived from non-cancerous cells have inhibitory effects on HCC. *CircWDR25* facilitated HCC cell proliferation and invasion by regulating the circWDR25/miR-4474-3p/arachidonate 15-lipoxygenase (ALOX15) and EMT axes, in which it enhances the expression of cytotoxic T-lymphocyte associated protein 4 (CTLA-4) in HSCs and programmed cell death 1 ligand 1 (PD-L1) in HCC cells, thereby influencing the immune response in the TME and ultimately affecting HCC prognosis and recurrence (Liu et al., 2022).

### 4.2 Exosomal circRNAs and tumor proliferation, metastasis, and invasion

Understanding the mechanisms of cell cycle regulation is crucial in studying the transformation of normal cells into malignant tumors, because abnormal cell proliferation plays a vital role in this process (Fernández et al., 2002). Researchers have discovered that prolonged exposure to arsenic trioxide increases the exosome levels of *circRNA-100284* and facilitates its transfer to normal LO-2 cells, in which the circRNA interacts with miRNA-217, leading to accelerated cell cycle progression and promotion of hepatocellular malignancy (Dai et al., 2018). Similarly, *circ-00213*6 is upregulated in HCC tissues and cells and can be transferred via cancer cell-derived exosomes. It promotes HCC cell proliferation, migration, and invasion via the miR-19a-3p/Rab GTPase YPT1 homolog (RAB1A) pathway, is associated with poor prognosis, and correlates positively with disease staging (Yuan et al., 2022). However, it's worth noting that the study lacked clinical validation.

Exosomal *has-circ-0061395* is also significantly upregulated in the serum of patients with HCC, and silencing its expression led to cell cycle arrest and promotion of apoptosis. It interacts with the 3′untranslated region of miR-877-5p, resulting in increased expression of phosphatidylinositol 3-kinase regulatory subunit gamma (PIK3R3), thereby promoting HCC cell proliferation, migration, invasion, and malignant behavior (Yu et al., 2021). *Circ-0072088* is widely enriched in exosomes derived from HCC cells and can be secreted into surrounding normal cells through blood circulation, promoting cell transfer. It has a high diagnostic value in patients with HCC (AUC: 0.899), and high levels of *circ-0072088* are associated with poor prognosis. Knocking out *circ-0072088* abolished the sponging of miR-375 and downregulated matrix metalloproteinase 16 (MMP-16) expression, thus inhibiting HCC cell invasion and metastasis (Lin et al., 2021). *CircANTXR1* is highly abundant in exosomes isolated from the serum of patients with HCC and plays a crucial role in promoting HCC cell proliferation, migration, invasion, and tumorigenicity. It directly interacts with miR-532-5p, which in turn regulates the expression of X-ray repair cross-complementing 5 (XRCC5) to facilitate HCC development and worsen prognosis. Inhibiting *circANTXR1* could potentially serve as a novel therapeutic approach for HCC and as an early diagnostic marker (Huang et al., 2021). Matrix metalloproteinase 2 (MMP2) is closely associated with metastasis and malignant tumor dissemination (Zhu et al., 2017a). A recent study found that exosomal *circMMP2* (*has-circ-0039411*) can be transmitted to normal liver cell line L02, acting as a ceRNA to enhance the expression of MMP2 by sequestering miR-136-5p, thereby promoting the metastasis of HCC cells (Liu et al., 2020). Conversely, suppressing the expression of *circMMP2* reversed the aggressive behavior of L02 cells, and its increased expression is associated with a lower overall survival rate in patients. Exosomal *circ-0006602* is specifically expressed in the plasma of patients with HCC and enhances the expression of proteins associated with tumor proliferation in HCC cell lines; its diagnostic performance surpasses that of AFP, with an AUC of 0.907 compared with 0.694 (Guo et al., 2021). Combining *circ-0006602* and AFP greatly improved the early detection rate of HCC (AUC: 0.942).

A previous study reported that cytoplasmic circRNA *Cdr1as* functions as an oncogene by targeting the miR-7/epidermal growth factor receptor (EGFR) axis to promote HCC development (Yang et al., 2017). Further investigations revealed that *Cdr1as* is highly abundant in exosomes derived from HCC cells, acting as a ceRNA to promote HCC cell proliferation and migration by sequestering miR-1270 and upregulating AFP expression. *In vitro* experiments also demonstrated that exosomes derived from HCC cells overexpressing *Cdr1as* can directly transfer to adjacent normal cells, enhancing their proliferation and invasion abilities (Su et al., 2019). Other animal experiments showed that exosomal circular RNA tubulin tyrosine ligase-like family member 5 (*circTTLL5*) promotes mouse hepatocellular carcinoma cell proliferation and metastasis through the miR-136-5p/KIAA1522 axis, thereby suppressing tumor growth. This suggests that blocking exosome-mediated *circTTLL5* transfer might be a therapeutic target for HCC (Liu et al., 2023a). *CircTMEM45A* (*has-circ-0066659*) acts as a sponge for miR-665, regulating the expression of downstream insulin-like growth factor 2 (IGF2) to promote cell migration *in vitro* and the occurrence of HCC *in vivo*, and its expression correlates with tumor size, TNM staging, and vascular invasion. Patients with low *circTMEM45A* expression have longer survival (Zhang et al., 2020c). Another study has revealed that *circPTGR1* is abundant in exosomes released by highly metastatic HCC cells, and the presence of high levels of *circPTGR1* enhances EMT processes in low or non-metastatic cells through its interaction with miR-449a, thereby disrupting TME homeostasis, and is closely related to the clinical staging of HCC (Wang et al., 2019a). *Has-circ-0004001*, *has-circ-0004123*, and *has-circ-007579*2 were found to be upregulated in HCC serum exosomes according to bioinformatic analysis, and they demonstrated high sensitivity and specificity in distinguishing between healthy controls and patients with HCC. When combined, these three circRNAs achieved an AUC of 0.885 for HCC diagnosis, indicating their potential as biomarkers (Sun et al., 2020). Furthermore, exosomal *circAKT3* is associated with a high risk of HCC recurrence and death. Patients with high *circAKT3* expression have lower overall survival and disease-free survival rates, suggesting a poor prognosis (Luo et al., 2020).

EMT plays a crucial role in HCC metastasis, and exosomal circRNAs have been found to promote HCC progression by influencing EMT processes (Liu et al., 2022). Overexpression of HUR, an RNA-binding protein involved in maintaining mRNA stability, has been associated with poor prognosis in HCC (Song et al., 2020). *Has-circ-0074854* is highly expressed in both HCC cells and tissues, and downregulation of its expression affects the stability of the HUR protein. This attenuates zinc finger E-box binding homeobox 1 (ZEB1) signal transduction in HCC cells, which suppresses malignant behaviors, including cell proliferation, migration, and EMT, and can induce M2 polarization in macrophages, thereby promoting HCC progression (Wang et al., 2021a). There are experiments have revealed that *has-circ-0004277* competes to bind with HUR, leading to reduced expression of zona occludens-1 (ZO-1) and upregulation of the EMT-related transcription factor ZEB1 to promote HCC progression (Zhu et al., 2020). Notably, exosomal *has-circ-0004277* can be used to diagnose HCC, with a diagnostic area AUC reaching 0.816 (Zhu et al., 2020).

### 4.3 The influence of tumor angiogenesis on exosomal circRNAs

Inducing neovascularization is a crucial step in the invasion and metastasis of HCC, and exosomes released by cancer cells play a significant role in stimulating the formation of new blood vessels through signaling between HCC cells and endothelial cells (Morse et al., 2019). In the stimulation of tumor angiogenesis, exosomes can directly interact with vascular endothelial growth factor (VEGF)/VEGF receptors or act as carriers to transport circRNAs that target downstream molecules, indirectly contributing to the generation of new blood vessels (Zhang et al., 2022b). Huang et al. discovered that *circRNA-100338* is highly abundant in exosomes of HCC cells with high metastatic potential, and the mechanism is that it can promote the proliferation of human umbilical vein endothelial cells (HUVECs) and regulates angiogenesis by interacting with neuro-oncological ventral antigen 2 (NOVA2), an RNA-binding protein associated with vascular development and lumen formation. Thus, the level of *circRNA-100338* in postoperative HCC serum can predict the occurrence of postoperative metastasis (Huang et al., 2020). Another study reported that exosomal *circCMTM3* directly binds to miR-3619-5p and targets the downstream SRY-box transcription factor 9 (SOX9) to promote the proliferation, migration, invasion, and angiogenesis of HUVECs, enhancing their survival capacity and inducing tumor growth. The level of exosomal *circCMTM3* also correlates positively with tumor stage and lymph node metastasis (Hu et al., 2021). Besides, exosomal *circPAK1* has also been found to promote angiogenesis and metastasis in HCC cells (Hao et al., 2022).

### 4.4 CircRNAs promote tumor immune escape

Cytokine Storm is involved in the occurrence and development of many diseases and may be one of the important causes of poor prognosis (Fajgenbaum and June 2020). Studies have found that circRNAs may be involved in the development of cytokine storms, for example, has-circ-0004812 acts on the JAK/STAT, STAT3 signaling pathway through hsa-mir-1287-5p/IL6R, RIG-I axis, and causes a in COVID-19 inflammatory responses (Mohammadisoleimani et al., 2022). Similarly, circRNAs may also contribute to HCC by promoting inflammatory cytokine storm processes. Tumor cells can modify their surface antigens and change the TME of the surrounding tissue, enabling them to evade recognition and attack by immune cells, which ultimately leads to tumor immune escape (Villalba et al., 2013). Programmed cell death receptor 1 (PD-1) is an inhibitory receptor expressed on the surface of active lymphocytes such as T cells, B cells, and NK cells (Greten and Sangro, 2017). Overexpression of PD-1 can lead to dysfunction of these cells and facilitate tumor immune evasion (Gao et al., 2009). In recent years, immune checkpoint blockade therapy, particularly anti-PD-1 therapy targeting the PD-1/PD-L1 axis and immune cell exhaustion, has been extensively used to treat various malignancies, including HCC. It has also been approved as a second-line treatment for advanced HCC (El-Khoueiry et al., 2017). However, less than 20% of patients with advanced HCC respond effectively to anti-PD-1 treatment because of innate or acquired resistance (Zhu et al., 2018). The specific mechanisms underlying resistance to anti-PD-1 immune therapy in HCC are still unclear. Nevertheless, some studies have demonstrated the involvement of exosomal circRNAs in regulating cancer immune evasion, promoting exhaustion of NK cells and CD8^+^ effector T cells, expanding regulatory T cells (Tregs), driving cancer progression, and inducing resistance to anti-PD-1 therapy (Chen et al., 2019).

NK cells play an important role in the antitumor immune response, and T cell immunoglobulin and mucin domain-containing protein 3 (TIM-3) is expressed on the surface of NK cells as an inhibitory molecule. Increased expression of TIM-3 is associated with NK cell dysfunction and death (Zheng et al., 2019). Exosomal *circUHRF1* (*has-circ-0048677*) derived from HCC cells can be delivered to NK cells in the body fluid circulation where it induces NK cell dysfunction and exhaustion by upregulating the expression of downstream target gene *TIM3* through adsorption of miR-449c-5p. This promotes cancer immune escape and drives resistance to anti-PD-1 immune therapy in patients with HCC (Zhang et al., 2020a). Therefore, analyzing the expression levels of *circUHRF1* can identify patients who are resistant to PD-1 therapy and improve the clinical efficacy in patients with HCC.

CD8^+^ effector T cells also play a key role in the immune system (Greten and Sangro, 2017). Recent studies have shown that *circCCAR1* is significantly enriched in exosomes derived from HCC and it promotes immune evasion of cancer cells and resistance to anti-PD-1 immune therapy by acting on the miR-127-5p/WT1 associated protein (WTAP) axis, increasing the stability of PD-1 protein, and causing dysfunction of CD8^+^ T cells (Hu et al., 2023). Additionally, circRNAs in exosomes derived from HCC cells, specifically *circGSE1*, act on the miR-324-5p/transforming growth factor beta receptor 1 (TGFBR1)/SMAD family member 3 (Smad3) axis to induce expansion of regulatory Tregs and facilitate immune evasion of HCC cells (Huang et al., 2022).

Macrophages can also influence the liver immune response in HCC (Tian et al., 2019). Exosomal circRNAs mediate intercellular communication between macrophages and HCC cells, thereby mediating HCC progression (Han et al., 2019). Depending on the stimuli, macrophages can polarize into two phenotypes: classical (M1) with anti-tumor properties or alternative (M2), with pro-tumor properties. During HCC progression, tumor cells often induce M2 polarization of macrophages to promote tumor growth (Komohara et al., 2016). Research has identified the overexpression of *circtMEM181* in HCC tissues, particularly in patients with a poor PD-1 response. The mechanism involves HCC cells delivering *circtMEM181* to macrophages via exosomes, where it binds to miR-488-3p and upregulates CD39 expression. This cooperative action between macrophages and HCC cells activates the ATP-adenosine pathway in the HCC TME, which interferes with the proliferation of CD8^+^ T cells, inducing CD8^+^ T cell exhaustion, and promoting resistance to PD-1 antibodies (Lu et al., 2021). As a result, the responsiveness to PD-1 therapy is limited. These findings highlight the potential of exosomal circRNAs in HCC immunotherapy and their potential as therapeutic targets. Furthermore, the ratio of Tregs to CD8^+^ T cells can serve as a predictive marker for the anti-tumor response to PD-1/PD-L1 inhibitors (Huang et al., 2022).

### 4.5 Exosomal circRNA and cancer metabolism

Malignant tumors rely on aerobic glycolysis to generate the substantial amount of energy required for the rapid proliferation of tumor cells; thus, glycolysis plays a significant role in the accelerated progression of malignant tumors (Lunt and Vander Heiden, 2011). Exosomal circRNAs have been found to influence the glycolytic process in HCC. The level of *CircFBLIM1* is significantly elevated in the serum exosomes of patients with HCC and HCC cells. It can be transferred to HCC cells through exosomes and acts on the miR-338/low-density lipoprotein receptor-related protein 6 (LRP6) axis, inducing the glycolytic process in HCC (Lai et al., 2023). Another study discovered that in HCC, exosomal *circ-ZNF652* can adsorb miR-29a-3p, leading to increased expression of the downstream target gene *GUCD1*. This, in turn, promotes cancer cell glycolysis and affects malignant phenotypes like proliferation, migration, and invasion. Silencing the expression of *circ-ZNF652* could inhibit HCC progression (Li et al., 2020).

### 4.6 The effects of exosomal circRNAs on cancer drug resistance

Drug resistance is a significant factor contributing to the poor prognosis of HCC, and the dysregulation of extracellular vesicle circRNAs has been identified to play a role in tumor drug resistance (Li et al., 2019). Sorafenib, a Food and Drug Administration (FDA)-approved first-line treatment for advanced HCC, has shown reduced efficacy in recent years because of the emergence of drug resistance, thus limiting its clinical utility (Wen et al., 2022; Zhu et al., 2017b). A study has revealed that *circRNA-SORE* interacts with Y-box binding protein 1 (YBX1) in the cytoplasm, inhibiting the translocation of YBX1 to the cell nucleus, preventing pre-mRNA processing factor 19 (PRP19)-mediated ubiquitination and nuclear degradation of YBX1, and consequently affecting the expression of downstream target genes controlled by YBX1 (including *AKT*, *RAF1* (encoding Raf-1 proto-oncogene), *ERK*, *MYC* (encoding c-Myc), and *TGFB1* (encoding transforming growth factor beta 1)). Silencing the expression of *circRNA-SORE* was demonstrated to enhance the efficacy of sorafenib (Xu et al., 2020a). Lenvatinib, a tyrosine kinase inhibitor, has been approved by the FDA as a targeted therapy drug for advanced HCC alongside sorafenib; however, drug resistance remains a significant obstacle to its clinical use (Personeni et al., 2019). In studies investigating lenvatinib resistance mechanisms, it was discovered that the levels of *circPAK*1 were significantly increased in extracellular vesicles derived from lenvatinib-resistant HCC cells. The primary mechanism involves *circPAK1* competitively binding to the cytoskeletal protein 14-3-3ζ, which promotes the nuclear translocation and inactivation of yes associated protein (YAP) and the Hippo signaling pathway (Hao et al., 2022). The Hippo signaling pathway is an inhibitory pathway primarily responsible for regulating cell size and tumor volume, with YAP serving as a downstream effector molecule. YAP, considered an oncoprotein, is regulated by phosphorylation and its subcellular localization. Phosphorylated YAP interacts with 14-3-3ζ, exits the cell nucleus, and undergoes ubiquitination and degradation mediated by beta-transducin repeat containing E3 ubiquitin protein ligase (β-Trcp). However, when the Hippo signaling pathway is inhibited, YAP remains non-phosphorylated, enters the cell nucleus through the nuclear membrane, interacts with transcription factors, and initiates the transcription of downstream target genes (Zhao et al., 2007). Furthermore, HCC-derived *circPAK1* can transfer from extracellular vesicles to lenvatinib-sensitive HCC cells, thereby reducing their drug sensitivity (Hao et al., 2022).

Cisplatin (DDP) is commonly used as a first-line chemotherapy drug for HCC; however, resistance to DDP has a significant impact on the prognosis of patients with HCC (Zhou et al., 2019). Previous studies have demonstrated that *CircZFR* promotes the progression of HCC by influencing the miR-511/AKT1 axis (Yang et al., 2019). Further investigation into its resistance mechanism revealed that *CircZFR* is highly expressed in HCC-associated fibroblasts and is abundant in their extracellular vesicles. Through these extracellular vesicles, *CircZFR* can be transferred to HCC cells, where it inhibits the signal transducer and activator of transcription 3 (STAT3)/nuclear factor kappa B (NF-kβ) signaling pathway, ultimately promoting HCC progression and chemotherapy resistance (Zhou et al., 2022). Transarterial chemoembolization (TACE) is a treatment option for patients with advanced HCCs (Elderkin et al., 2023). In a recent study, researchers examined the variation in the circRNA contents of exosomes during TACE treatment and found that the abundance of exosomal *circ-G004213* increased after TACE treatment compared to before treatment. This circRNA acts as a sponge for miR-513b-5p and enhances the expression of pre-mRNA processing factor 39 (PRPF39), thus improving the sensitivity of HepG2 cells to cisplatin and improved survival *in vivo*. Besides, the *circ-G00421* expression level correlated positively with post-TACE prognosis, it may be used as an indicator for predicting the efficacy of TACE (Qin et al., 2021). This research provides a new potential combination treatment strategy to overcome chemotherapy resistance in HCC.

## 5 Conclusion

**FIGURE 2:**
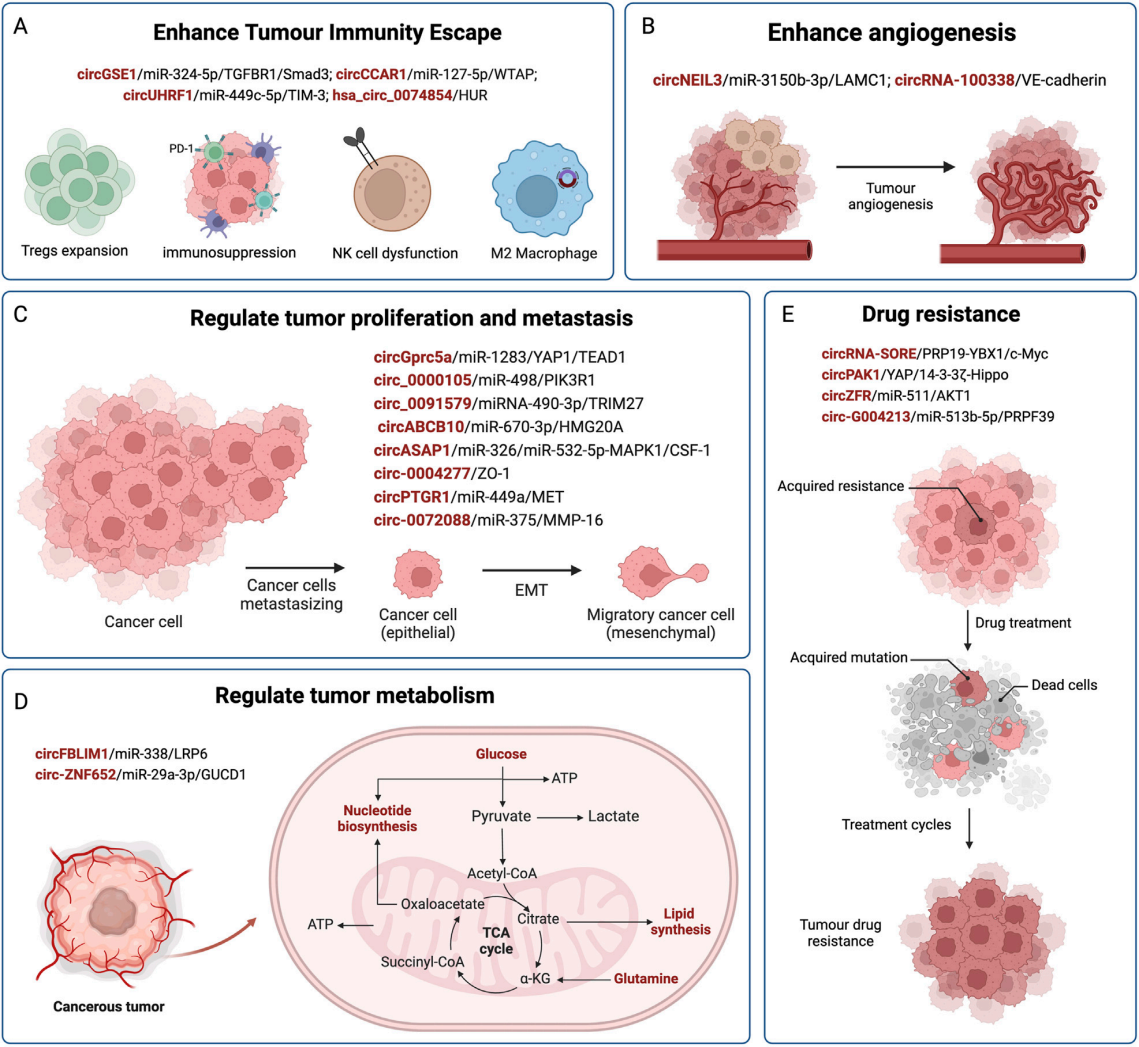
The regulatory role of exosomal circRNAs in HCC. CircRNAs are delivered by exosomes from donor cells to recipient cells, and affect the progression of HCC by competitively binding miRNAs and acting on downstream target genes. Exosomal circRNAs are involved in mediating the malignant phenotype of HCC, such as immune evasion **(A)**, angiogenesis **(B)**, HCC cell proliferation and metastasis **(C)**, metabolism **(D)**, and drug resistance **(E)**.

It is predicted that by 2040, there will be 1.3 million deaths from HCC, an increase of over 50% compared with that in 2020 (Rumgay et al., 2022). In recent years, an increasing number of exosomal circRNAs have been found to exhibit significantly changed expression profiles in HCC. The abnormal expression of circRNAs plays an important role in the occurrence and development of HCC. They transfer and transmit biological information between normal cells and HCC cells through exosomes, affecting malignant phenotypes such as HCC cell proliferation, invasion and metastasis, immune escape, cellular metabolism, and drug resistance (as shown in Figure 2; Table 1). Pathological diagnosis remains the gold standard for HCC; however, its early clinical application is limited by invasive procedures. Exosomal circRNAs, as novel biomarkers, exist in various body fluids, with high abundance, stability, and sensitivity, and can be sampled in a relatively non-invasive manner. They can serve as biomarkers for the early diagnosis and prognostication of HCC, as well as representing new therapeutic targets to improve the prognosis of patients with HCC. Based on all the facts about the circRNA in pathophysiology of cell proliferation suggest that some of the biological functions govern by genes via noncoding circRNA in immunocompromised beings. Moreover, current research on exosomes is still in its nascent phase, and there are numerous hurdles to overcome before their clinical application can guide practical treatments, including the efficient isolation and extraction of exosomes. Nevertheless, we maintain that exosomal circRNAs will undoubtedly play a pivotal role in the future diagnosis and treatment of HCC.

**TABLE 1:** Exosomal circRNAs involved in HCC.

Number	CircRNAs	Expression change	Exosome source	Mechanism	Influence phenotype	Clinical application	References
1	hsa-circ −0028861	up	serum	—	—	diagnosis	Wang et al. (2021b)
2	hsa-circ-0070396	up	plasma	—	—	diagnosis	Lyu et al. (2021)
3	hsa-circ-0051443	down	plasma Huh7 Hep3B	miR-331-3p/BAK1	apoptoscell (−) cell cycle (+)	diagnosis prognosis therapy	Chen et al. (2020)
4	hsa-circ-0004658	up	SMMC-7721	miR-499b-5p/JAM3	proliferation (−), apoptosis (+)	diagnosis therapy	Zhang et al. (2022a)
5	hsa-circ-004310 (circ-WDR25)	up	HSCs	miR-4474-3p/ALOX-15	proliferation (+) migration (+), invasion (+), EMT process (+)	therapy prognosis	Liu et al. (2022)
6	circRNA-100284	up	serum	miR-217/EZH2	Cell cycle (+) proliferation (+)	therapy	Dai et al. (2018)
7	circ-002136	up	Huh7 HA22T	miR-19a-3p/RAB1A	proliferation (+), migration (+) invasion (+), apoptoscell (−)	diagnosis therapy prognosis	Yuan et al. (2022)
8	hsa-circ-0061395	up	serum SNU-387, Huh7	miR-877-5p/PIK3R3	proliferation (+), migration (+), invasion (+) apoptoscell (−)	therapy prognosis	Yu et al. (2021)
9	circ-0072088	up	HCC cells, plasma huh-7	miR-375/MMP-16	proliferation (+), migration (+), invasion (+)	diagnosis prognosis	Lin et al. (2021)
10	circANTXR1	up	serum	miR-532-5p/XRCC5	proliferation (+), migration (+), invasion (+)	diagnosis therapy	Huang et al. (2021)
11	hsa-circ-0039411 (circ-MMP2)	up	97H LM3	miR-136-5p/MMP2	migration (+)	therapy	Liu et al. (2020)
12	circ-0006602	up	HCC-97L HCC-LM3 HepG2	—	proliferation (+), migration (+), invasion (+)	diagnosis	Guo et al. (2021)
13	circRNA Cdr1as	up	HepG2 SMMC-7721	miR-1270/AFP	proliferation (+), migration (+)	therapy	Yang et al. (2017), Su et al. (2019)
14	circTTLL5	up	—	miR-136-5p/KIAA1522	proliferation (+), migration (+)	therapy	Liu et al. (2023a)
15	circTMEM45A	up	plasma	miR-665/IGF2	migration (+), invasion (+)	prognosis diagnosis	Zhang et al. (2020c)
16	circPTGR1	up	serum HepG2 SMCC-7721 L-02 HEP3B MHCC 97-L/H HCC-LM3	miR-449a/EMT	migration (+), invasion (+)	prognosis therapy	Wang et al. (2019a)
17	hsa-circ-0004001	up	serum	—	—	diagnosis	Sun et al. (2020
18	hsa-circ-0004123	up	serum	—	—	diagnosis	Ibid
19	hsa-circ-0075792	up	serum	—	—	diagnosis	Ibid
20	circAKT3	up	serum	—	occurrence (+), metastasis (+)	prognosis	Luo et al. (2020)
21	hsa-circ-0074854	up	HepG2	HuR	macrophage M2 activation (+)	therapy	Wang et al. (2021a)
22	Hsa-circ-0004277	up	plasma SMMC-7721 HepG2	HuR	proliferation (+) migration (+), invasion (+), EMT process (+)	diagnosis	Zhu et al. (2020)
23	circRNA-100338	up	plasma Hep3B HLE Huh7 BEL7402SMCC7721 MHCC97L MHCC97H HCCLM3 HCCLM6	—	migration (+) angiogenesis (+)	prognosis	Huang et al. (2020)
24	circCMTM3	up	serum Hep3B	miR-3619-5p/SOX9	angiogenesis (+) proliferation (+) migration (+), invasion (+)	therapy, diagnosis	Hu et al. (2021)
25	circPAK1	up	LM3-LR Hep-3B-LR (levaritinib resistance)	14-3-3ζ/YAP	proliferate (+), migration (+), invasion (+) angiogenesis (+) apoptoscell (−), levaritinib, resistance (+)	therapy	Hao et al. (2022)
26	circUHRF1	up	serum HepG2 HCCLM3SMMC-7721 Huh7 PLC/PRF/5 Hep3B	6 miR-449c-5p/TIM-3	Induces NK cell death and resistance to PD-1 therapy	therapy, diagnosis	Zhang et al. (2020a)
27	circCCAR1	up	serum	miR-127-5p/WTAP	proliferation (+), migration (+), Promotes PD-1 resistance and inhibit immunity	therapy, diagnosis	Hu et al. (2023)
28	circGSE1	up	Huh7 HepG2	miR-324-5p/TGFBR1/Smad3	Tregs cell proliferation (+), proliferation (+), migration (+), invasion (+)	therapy	Huang et al. (2022)
29	circTMEM181	up	Huh-cell lines	miR-488-3p/CD39	Induces macrophage dysfunction, forming a suppressive tumor microenvironment	therapy	Lu et al. (2021)
30	circFBLIM1	up	serum SNU-387 Huh7	miR-338/LRP6	glycolysis (+)	therapy	Lai et al. (2023)
31	circ-ZNF652	up	serum SNU-387 Huh7	miR-29a-3p/GUCD1	proliferate (+), migration (+), invasion (+), glycolysis (+)	therapy	Li et al. (2020)
32	circRNA-SORE	up	HepG2-SR HepG2-P	YBX1	resistance to sorafenib (+)	therapy	Xu et al. (2020a)
33	circPAK1	up	LM3-LR Hep-3B-LR (levaritinib resistance)	14-3-3ζ/YAP	proliferate (+), migration (+), invasion (+), angiogenesis (+), apoptoscell (−), levaritinib resistance (+)	therapy	Hao et al. (2022), Zhao et al. (2007)
34	circZFR	up	Huh7 MHCC97L (cisplatin resistance)	7	proliferation (+), migration (+), invasion (+), cisplatin resistance (+)	therapy, prognosis	Yang et al. (2019), Zhou et al. (2022)
35	hsa-circ-G004213	up	plasma HepG2	miR-513b-5p/PRPF39	cisplatin resistance (+)	therapy, prognosis	Qin et al. (2021)

One potential strategy to enhance the survival rate of patients with (HCC) is effectively delivering drugs into tumor cells. Exosomes, owing to their unique characteristics, can serve as cargo carriers for various functional molecules, including RNA, proteins, lipids, and circRNAs. Exploiting this feature, researchers have explored the use of nanotechnology to harness exosomes as vehicles to deliver drugs and functional RNAs, enabling targeted and precise tumor therapy (Ha et al., 2016). In recent years, RNA-based targeted therapy has emerged as a promising approach in cancer treatment, and circRNAs are anticipated to be valuable biomarkers for diagnosing, treating, and prognosticating liver cancer, particularly when they are encapsulated within exosomes. We believe that the combined utilization of circRNAs within exosomes and traditional biomarkers could significantly enhance their clinical utility in liver cancer, providing precise and personalized treatments for patients with HCC, ultimately leading to improved prognosis.
